# Association of self-reported sedentary time with insulin resistance among Korean adults without diabetes mellitus: a cross-sectional study

**DOI:** 10.1186/s12889-018-6237-4

**Published:** 2018-12-04

**Authors:** Kyeong Seok Kim, Seong Jun Kim, Seonggwan Kim, Dong-Woo Choi, Yeong Jun Ju, Eun-Cheol Park

**Affiliations:** 1 Premedical Courses, College of Medicine, Yonsei University, Seoul, Republic of Korea; 2 Department of Public Health, Graduate School, Yonsei University, Seoul, Republic of Korea; 3 Institute of Health Services Research, Yonsei University College of Medicine, 50 Yonsei-ro, Seodaemun-gu, Seoul, 03722 Republic of Korea; 4 Department of Preventive Medicine, Yonsei University College of Medicine, Seoul, Republic of Korea; 5 Present address: Department of Preventive Medicine and Public Health, Ajou University School of Medicine, Suwon, Republic of Korea; 6 Department of Preventive Medicine & Institute of Health Services Research, Yonsei University College of Medicine, 50 Yonsei-ro, Seodaemun-gu, Seoul, 120-752 Korea

**Keywords:** Insulin resistance, HOMA-IR, Sedentary time

## Abstract

**Background:**

A more sedentary lifestyle can result in insulin resistance. However, few research studies have assessed the association between insulin resistance and sedentary lifestyle in Asian populations. Therefore, this study aimed to investigate the association of sedentary time with insulin resistance. In addition, we also investigate the moderate effect of employment status, moderate-to-vigorous physical activity (MVPA), and body mass index (BMI) in this association.

**Methods:**

Data from 2573 individuals who participated in the 2015 Korean National Health and Nutrition Examination Survey were analyzed. Sedentary time was measured using self-administered questionnaires, and IR data were estimated using the homeostasis model assessment–insulin resistance index (HOMA-IR). Adjusted odds ratio (OR) and 95% confidence intervals (CIs) from a multivariable logistic regression model were generated for all participants. Subgroup analysis was only performed between sedentary time and HOMA-IR stratified by employment status, because moderate effects were not significant in the tests for interaction for MVPA and BMI. For all analyses, the individuals were categorized as having high or normal HOMA-IR values (> 1.6 and ≤ 1.6, respectively).

**Results:**

A HOMA-IR > 1.6 was observed in 40.3% of the sedentary time Q1 (low) group (< 5.0 h/day), 41.4% of the sedentary time Q2 (middle-low) group, 44.2% of the sedentary time Q3 (middle-high) group, and 48.4% of the sedentary time Q4 (high) group (≥10.0 h/day). When the low level sedentary time group was used as the reference group, the high level sedentary time group was significantly associated with high IR value (HOMA-IR > 1.6) (OR = 1.40, 95% CI: 1.060–1.838). However, this association was not significant across the other sedentary time groups. Moreover, participants reporting a high sedentary time and were employed had 1.67 times the odds of having a high IR value (HOMA-IR > 1.6) compared to those who reported having a low sedentary time and were employed (OR = 1.67, 95% CI: 1.184–2.344). In the unemployed participants, sedentary time was not associated with IR.

**Conclusions:**

High sedentary time (≥10.0 h/day) was associated with elevated HOMA-IR among Korean adults without diabetes mellitus. Furthermore, the association between high sedentary time and HOMA-IR values was more pronounced in the employed population.

## Background

Insulin resistance (IR) occurs when the body’s response to insulin is lower than normal. The deterioration of insulin makes the cells unable to burn glucose effectively, which causes the body to over-produce insulin and contribute to the occurrence of various diseases [1, 2]. IR plays a key role in the development of type 2 diabetes and contributes to the pathophysiology of burdensome disease including obesity, metabolic syndrome, and cardiovascular disease. IR is commonly considered an important clinical and biochemical determinant and has been a subject of interest, as it has effects on various chronic disease such as diabetes, cardiovascular disease, hypertension, and metabolic syndrome [3]. For example, previous studies have reported that family history of type 2 diabetes mellitus (T2DM), non-alcoholic fatty liver disease, obesity, lack of exercise, high triglyceride levels, low levels of high-density lipoprotein, high-molecular weight (HMW)-adiponectin levels, hepatitis C, hemochromatosis, or hypercortisolism are associated risk factors [4–9]. Studies on insulin resistance have been reported in neuroscience and clinical research fields. Studies on insulin resistance reported that IR is associated with cognitive dysfunction such as cognitive decline and cognitive impairment [10].

Meanwhile, recent studies have focused on factors such as lack of exercise and a sedentary lifestyle in relation to insulin resistance [11]. Studies have reported that poor physical activity status is associated with insulin resistance, with attention being focused on sitting time which is directly related to physical activity status [12]. Recently, the need to investigate the relationship between sitting time and health status is increasing, and it is also necessary to establish a basis for this research. Sedentary time has a significant effect on health, and individuals who use more screen-based entertainment have a higher risk of clinically confirmed cardiovascular disease events [13]. Furthermore, a cohort study of people from Hawaii and California revealed that sedentary time was associated with cancer mortality [14]. A meta-analysis from 2015 revealed that sedentary time was associated with cardiovascular disease and all-cause mortality [15], and another meta-analysis from 2012 also demonstrated that sedentary time was associated with various diseases including type II diabetes [16].

In addition, few research have evaluated the association between sedentary time and IR in the Korean population, and most studies regarding sedentary lifestyle and diabetes during 2012–2015 only evaluated non-Asian populations [17, 18]. To fill this research gap, there is a need to investigate studies on this topic in Asian population. Therefore, the purpose of this study was to investigate the association of sedentary time with insulin resistance.

Meanwhile, physical activity and BMI are well-known factors that are independently associated with insulin resistance [19–21]. In addition, as second longest working hours in OECD countries, high sedentary time are becoming a lot of controversy in Korea. Hence, we also investigate the moderate effect of employment status, moderate-to-vigorous physical activity (MVPA), and body mass index (BMI) in this association.

## Methods

### Study population

**Fig. 1:**
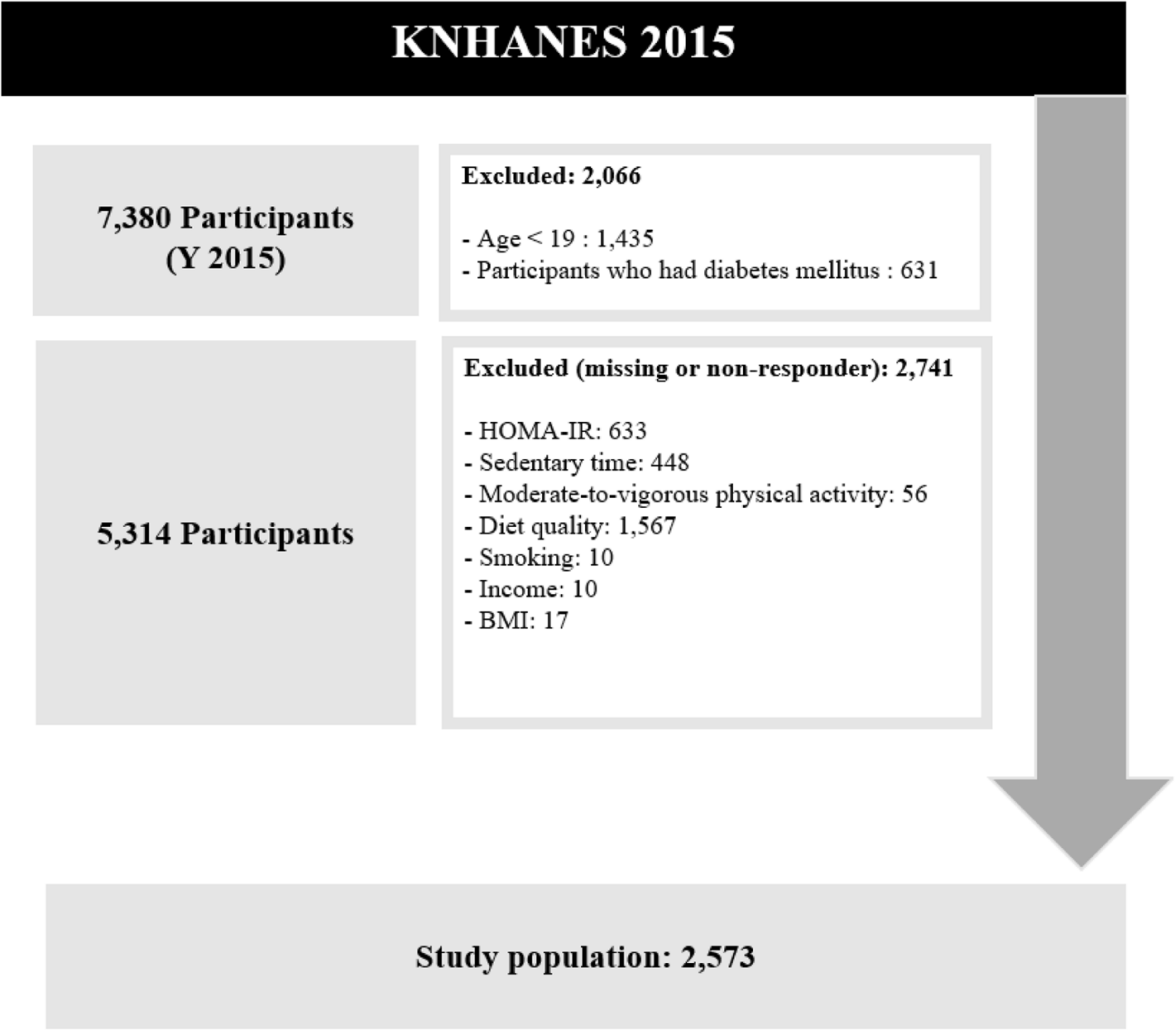
Is the flow diagram of the study participants for present study

The present study evaluated data from the 2015 Korean National Health and Nutrition Examination Survey (KNHANES), which was performed by the Korean Center for Disease Control. A cross-sectional survey, it is a multistage, stratified area probability sample of civilian non-institutionalized Korean households by geographic area, age, and gender groups. This survey is composed of three parts—a health interview, health examination, and nutrition survey—all of which were performed by trained medical staff and dieticians. A total of 7380 individuals participated in the 2015 KNHANES. However, the present study excluded participants with diabetes (fasting blood glucose levels > 126 mg/dL, or physician-diagnosed diabetes mellitus), in order to avoid confounding the IR-related analyses [22]. In addition, participants were excluded if they were missing data and if they were aged < 19 years. Any respondents who did not provide data on sedentary time, moderate-to-vigorous physical activity (MVPA), subjective health status, age, income, employment status, education, stress, smoking, drinking, marriage status, BMI, menopause, or who were aged < 19 years were excluded from the study (see details in Fig. 1).

The KNHANES data are openly available at the KNHANES website: https://knhanes.cdc.go.kr/knhanes/eng/index.do. Ethical approval for this study was granted by the institutional review board (IRB) of the KCDC Seoul, Korea (IRB #: 2015-01-02-6C).

### Measures

#### HOMA-IR

The dependent variable was defined as the homeostasis model assessment–insulin resistance index (HOMA-IR), which was calculated using the following formula:

$$ HOMA- IR=\frac{\mathrm{fasting}\ \mathrm{plasma}\ \mathrm{glucose}\left(\mathrm{mg}/\mathrm{dL}\right)\times \mathrm{fasting}\ \mathrm{plasma}\ \mathrm{insulin}\left(\upmu \mathrm{IU}/\mathrm{mL}\right)\ }{405} $$

Data regarding fasting plasma glucose and insulin levels were obtained from the 2015 KNHANES health examination results. For the present study, individuals were categorized as having high or normal HOMA-IR values (> 1.6 vs. ≤1.6, respectively) based on the Japanese Diabetes Society guidelines [23].

#### Sedentary time

The independent variable was defined as sedentary time. Sedentary time was estimated using the Global Physical Activity Questionnaire (GPAQ), and assessed via the following question: “How much time do you spend sitting or lying down during at work, at home, when travelling from place to place or when meeting friends, but excluding sleeping hours?”. Responses were divided into 4 categories using quartiles, with the Q1 group having < 5 h/day, the Q2 group having 5–7.9 h/day, the Q3 group having 8–9.9 h/day, and the Q4 group having ≥10 h/day. The validity of the GPAQ tool for Koreans was 0.79, which was reported to be sufficiently valid for the use of the tool [24].

#### MVPA

MVPA was measured using the GPAQ. GPAQ, developed by the World Health Organization (WHO), is a questionnaire measuring the amount of physical activity (occupation, mobility, leisure activity) and is a standardized questionnaire currently used in 50 countries. This questionnaire was developed and validated by the WHO to systematically monitor global physical activity levels as one of the main lifestyle disease risk factors. The validity of the GPAQ tool for Koreans was 0.64, which was reported to be sufficiently valid for the use of the tool [24]. Regarding MVPA, responses were divided into 2 categories (“yes” or “no”), with the “yes” group consisting of individuals who exercise moderately more than 2 hours and 30 minutes per week or intensively more than 1 hour and 15 minutes per week. 1 minute of intensive physical activity was defined as equivalent to 2 minutes of moderate physical activity.

#### Diet quality

To assess the diet quality, the mean adequacy ratio (MAR) index was calculated using the nutrient adequacy ratio (NAR) index. The NAR was calculated for each of nine nutrients (protein, vitamin A, thiamine, riboflavin, niacin, Ca, P, Fe, and vitamin C), whose recommended intake was set according to the dietary reference intakes for Koreans [25], using the following formula:

$$ \mathbf{NAR}={\mathrm{Participant}}^{\hbox{'}}\mathrm{s}\ \mathrm{daily}\ \mathrm{intake}\ \mathrm{of}\ \mathrm{a}\ \mathrm{nutrient}/\mathrm{recommended}\ \mathrm{nutrition}\ \mathrm{intake} $$

$$ \mathbf{MAR}=\Sigma \mathrm{NAR}/\mathrm{number}\ \mathrm{of}\ \mathrm{nutrients}. $$

NARs were truncated at 1 if the value was over 1. The MAR provides an index of the overall diet quality. A high MAR implies a high-quality diet [26].

#### Covariates

The analyses were adjusted for covariates that might be associated with HOMA-IR. These covariates were defined as age (19–29 years, 30–49 years, 50–69 years, and ≥ 70 years), sex, body mass index (BMI; underweight: < 18.5 kg/m^2^, normal: 18.5–24.9 kg/m^2^, and obese: ≥25.0 kg/m^2^), education level (elementary school or less, middle school, high school, and university or higher), income (monthly income quartiles), employment status (employed vs. unemployed or economically inactive), marital status (no vs. yes), subjective health status (good, normal, and bad) stress (no vs. yes), smoking status (current smoker, previous smoker, and never smoker), alcohol consumption (not in the last year, < 4 times per month, 2–3 time per week, and 4 times per week), and MVPA (no vs. yes).

### Statistical analysis

We first examined the distribution of each categorical variable. The chi-square test was used to calculate the distribution of each categorical variable and to confirm significant differences between groups. In addition, to produce an unbiased national estimate, a sample weight was assigned for the participating individuals to represent the Korean population. The sampling weight was calculated by accounting for the complex survey design, survey nonresponse, and post-stratification. Next, to investigate the association of sedentary time with insulin resistance, multivariable logistic regression analysis was used. In multivariable logistic regression, confounding variables such as age, sex, income, education level, employment status, marriage status, perceive health status, stress, smoking, alcohol intake, BMI, MVPA, and diet quality were controlled. To consider the considerable effect of employment status, MVPA, and BMI on sedentary behavior which has been reported in previous literature [27], we also examined whether employment status, MVPA, and BMI modified the association between sedentary time and the insulin resistance by introducing an interaction terms in the models. Then, subgroup analysis was only performed between sedentary time and HOMA-IR stratified by employment status, because moderate effects were not significant in the tests for interaction for MVPA and BMI variables.

All statistical analyses were performed using SAS software (version 9.4; SAS Institute, Cary, NC, USA), and differences with a *p*-value < 0.05 were considered statistically significant.

## Results

### Characteristics of the participants

In our study, 2573 participants were included to access the association between sedentary time and HOMA-IR. Table 1 shows the characteristics of the study population. Among the study population, 19.9% (*n* = 511) were in the sedentary time Q1 (low) group (< 5.0 h/day), 38.7% (*n* = 997) were in the sedentary time Q2 (middle-low) group (5.0–7.9 h/day), 22.4% (*n* = 577) were in the sedentary time Q3 (middle-high) group (8.0–9.9 h/day), and 19.0% (*n* = 488) were in the sedentary time Q4 (high) group (≥10.0 h/day). High IR values (HOMA-IR > 1.6) were observed for 40.3% (*n* = 206) of the sedentary time Q1 (low) group, 41.4% (*n* = 413) of the sedentary time Q2 (middle-low) group, 44.2% (*n* = 255) of the sedentary time Q3 (middle-high) group, and 48.4% (*n* = 236) of the sedentary time Q4 (high) group.

**Table 1:** General characteristics of study population

Variables	Total		HOMA-IR > 1.6				*P* Value
			YES		NO		
	N	%	N	%	N	%	
Sedentary time (hours per day)							0.0360
Q1 (< 5)	511	19.9	206	40.3	305	59.7	
Q2 (5~7.9)	997	38.7	413	41.4	584	58.6	
Q3 (8~9.9)	577	22.4	255	44.2	322	55.8	
Q4 (≥10)	488	19.0	236	48.4	252	51.6	
Age							0.0010
19~29	488	19.0	225	46.1	263	53.9	
30~39	508	19.7	203	40.0	305	60.0	
40~49	623	24.2	243	39.0	380	61.0	
50~59	679	26.4	294	43.3	385	56.7	
≥60	275	10.7	145	52.7	130	47.3	
Sex							0.0001
Male	1006	39.1	481	47.8	525	52.2	
Female	1567	60.9	629	40.1	938	59.9	
Income							0.1138
Low	566	22.0	261	46.1	305	53.9	
Middle low	648	25.2	276	42.6	372	57.4	
Middle high	684	26.6	305	44.6	379	55.4	
High	675	26.2	268	39.7	407	60.3	
Education level							0.0073
Elementary school or less	212	8.2	112	52.8	100	47.2	
Middle school	226	8.8	89	39.4	137	60.6	
High school	1023	39.8	453	44.3	570	55.7	
University or more	1112	43.2	456	41.0	656	59.0	
Employment status							<.0001
Employed	1748	67.9	748	42.8	1000	57.2	
Unemployed or economically inactive	825	32.1	362	43.9	463	56.1	
Marriage status							0.2802
Unmarried	597	23.2	269	45.1	328	54.9	
Married	1976	76.8	841	42.6	1135	57.4	
Perceive health status							0.0006
Healthy	873	33.9	332	38.0	541	62.0	
Normal	1322	51.4	597	45.2	725	54.8	
Unhealthy	378	14.7	181	47.9	197	52.1	
Stress							0.0060
No	1807	70.2	748	41.4	1059	58.6	
Yes	766	29.8	362	47.3	404	52.7	
Smoking							0.0017
None smoker	1660	64.5	675	40.7	985	59.3	
Previous smoker	471	18.3	232	49.3	239	50.7	
Current smoker	442	17.2	203	45.9	239	54.1	
Alcohol intake							0.7786
No	539	20.9	243	45.1	296	54.9	
< 4 times a month	1545	60.1	657	42.5	888	57.5	
2~3 times a week	374	14.5	160	42.8	214	57.2	
≥4 times a week	115	4.5	50	43.5	65	56.5	
BMI							<.0001
Underweight (BMI < 18.5)	114	4.4	12	10.5	102	89.5	
Normal (18.5 ≤ BMI < 25)	1666	64.8	533	32.0	1133	68.0	
Obesity (25 ≤ BMI)	793	30.8	565	71.3	228	28.7	
Moderate-to-vigorous physical activity							0.1828
No	1213	47.1	540	44.5	673	55.5	
Yes	1360	52.9	570	41.9	790	58.1	
Diet quality (Mean ± S.D)	0.83 ± 0.16		0.83 ± 0.16		0.83 ± 0.16		0.8722
Total	2573	100.0	1110	43.1	1463	56.9	

### Multivariable logistic regression results of association between ST and HOMA-IR

Table 2 show the results of the multivariable logistic regression analysis for the association between sedentary time and HOMA-IR. High level of sedentary time (≥10 h/day) was significantly associated with high IR value (HOMA-IR > 1.6) (OR = 1.40, 95% CI: 1.060–1.838).

**Table 2:** Multivariable logistic regression analysis of the association between sedentary time and HOMA-IR

Variables	HOMA-IR > 1.6			
	Adjusted-OR^a^	95% CI		
Sedentary time (hours per day)				
Q1 (< 5)	1.00			
Q2 (5~7.9)	1.09	0.862	–	1.365
Q3 (8~9.9)	1.20	0.900	–	1.606
Q4 (≥10)	1.40	1.060	–	1.838

Notes: ^a^Adjusted odds ratios (OR) were calculated using logistic regression analysis and adjusted for age, sex, income, education level, employment status, marriage status, perceive health status, stress, smoking, alcohol intake, BMI, moderate-to-vigorous physical activity, and diet quality

### Sub-group analysis results by employment status

The subgroup analysis results are shown in Table 3. Subgroup analysis was only performed between sedentary time and HOMA-IR stratified by employment status, because moderate effects were not significant in the tests for interaction for MVPA and BMI variables (MVPA: *p* for interaction, *p* = 0.2679; BMI: *p* for interaction, *p* = 0.2003). Subgroup analysis showed significant differences in employment status (*p* for interaction: *p* = 0.0217). Participants reporting high sedentary time and were employed had 1.67 times the odds of having a high IR value (HOMA-IR > 1.6) compared to those who reported having a low sedentary time and were employed (OR = 1.67, 95% CI: 1.184–2.344). In the unemployed participants, sedentary time was not associated with IR.

**Table 3:** Subgroup analysis of the association between sedentary time and HOMA-IR by employment status

Variables		HOMA-IR > 1.6			
		Adjusted-OR^a^	95% CI		
Employment status	Sedentary time				
Employed	Q1 (< 5)	1.00			
	Q2 (5~7.9)	1.22	0.928	–	1.597
	Q3 (8~9.9)	1.24	0.872	–	1.769
	Q4 (≥10)	1.67	1.184	–	2.344
Unemployed or economically inactive	Sedentary time				
	Q1 (< 5)	1.00			
	Q2 (5~7.9)	0.85	0.530	–	1.353
	Q3 (8~9.9)	1.11	0.658	–	1.864
	Q4 (≥10)	0.87	0.511	–	1.491

Notes: ^a^Adjusted odds ratios (OR) were calculated using logistic regression analysis and adjusted for age, sex, income, education level, marriage status, perceive health status, stress, smoking, alcohol intake, BMI, moderate-to-vigorous physical activity, and diet quality

## Discussion

Although there are many studies that showed that high sedentary time was negatively associated with health outcomes, little research has been conducted on this in Korea. Therefore, there is a growing interest in researching sedentary time. Thus, we investigate the association of sedentary time with IR in Korean adults without diabetes mellitus. In addition, we also investigate the moderate effect of employment status, MVPA, and BMI in this association. Given that these issues remain a concern, it is necessary to design effective strategies to prevent and manage reduced insulin resistance and its negative health outcomes.

Through multivariable analysis, our findings revealed that sedentary time was associated with IR among an adult population without diabetes mellitus. This was consistent with previous studies. Cross-sectional analysis with 4757 adults in the United States of America reported that higher amounts of sedentary time was associated with higher HOMA-IR [28]. Another study that included 2027 young adult participants (aged 38–50 years) also confirmed that having higher amounts of sedentary time was cross-sectionally associated with higher HOMA-IR [29]. However, other studies reported that having higher amounts of sedentary time was not cross-sectionally associated with HOMA-IR or fasting insulin [30, 31]. The differences in results might be explained by the differences between objectively measured time and small sample sizes.

Regarding subgroup analysis, it was only performed between sedentary time and HOMA-IR stratified by employment status, because moderate effects were not significant in the tests for interaction for MVPA and BMI variables. As the interaction tests proved to be statistically significant, we confirmed that the association between sedentary time with HOMA-IR values was more pronounced in the employed population. For most working adults, time spent sitting in the workplace is likely a greater contributor to overall sitting time [32]. In addition, studies reported that “work” was more sedentary and had less light-intensity activity than “non-work” [33, 34]. Hence, the association between sedentary time and HOMA-IR in workers may be prominent due to prolonged sedentary time in work life. Therefore, there is a need to concentrate efforts to efficiently manage sedentary time, especially for workers. Recently, in an attempt to tackle the country’s notoriously long work hours, South Korea officially dropped its maximum work week to 52 h in July 2018 in an effort to improve the quality of life among its citizens. As following strategies, approaches to effectively manage workers’ own sedentary time during working hours should also be considered. Strategies should be supported to manage the sedentary time efficiently, such as providing support for the conditions for the physical activity of the workers in the workplace. Based on the lessons learned from many prior programs that aimed at efficient sedentary time management at the workplace, policy makers should consider efficient strategies to manage the sedentary time in the workplace [35].

The present study has several limitations that warrant consideration. First, individuals with diabetes were excluded because the vast majority of these patients receive diabetes treatments that can alter insulin sensitivity and HOMA-IR values. Although this approach is useful for data cleaning, it precludes any analysis of the association between sedentary time and HOMA-IR values among patients with diabetes, and further studies are needed to evaluate this issue. Second, the present study was unable to identify a causal relationship between sedentary time and insulin resistance because the study design was cross sectional and information was obtained via self-reported. Thus, prospective cohort studies or prospective clinical research studies are needed to examine the causal relationships between sedentary time, employment status, and HOMA-IR values. Third, it is possible that IR can change over time, even in cases with controlled sedentary time. For example, a previous study [36] revealed that interrupting sedentary time with short walks was associated with lower postprandial glucose and insulin levels among overweight/obese adults. Fourth, we could not measure mobility impairment that have been associated with sedentary time, due to limitations of data. This probably unreported population characteristic could have influenced the association between sedentary time and insulin resistance.

## Conclusion

In conclusion, this study revealed that only high sedentary time (≥10.0 h/day) was associated with HOMA-IR among adults Korean without diabetes mellitus. However, this association was not significant across the other sedentary time groups. Furthermore, the association of high sedentary time (≥10.0 h/day) with HOMA-IR values was more pronounced in the employed population.

## Abbreviations

BMI: Body Mass Index
GPAQ: Global Physical Activity Questionnaire
HOMA-IR: Homeostasis Model Assessment-Insulin Resistance
IR: Insulin Resistance
MAR: Mean Adequacy Ratio
MVPA: Moderate-to-Vigorous Physical Activity
NAR: Nutrient Adequacy Ratio
